# Mr. Hume on His Test for Arsenic

**Published:** 1812-08

**Authors:** Jos. Hume

**Affiliations:** Long Acre


*To the Editors of the Medical and Physical Journal.*

GENTLEMEN,

WHEN first I published my *Test for Arsenic*, in the Philosophical Magazine for May 1809, I had made no other experiments to ascertain its powers, compared with the usual methods, than just to satisfy myself that it surpassed all the tests at that time known; being infinitely more correct, delicate, and practicable, in all respects.

The great superiority of this test, the *combining of silver with the arsenic*, is now perfectly confirmed by the joint labors of doctors Marcet and Roget; so far, indeed, that these gentlemen have conferred a lasting benefit on society, done honor to themselves, and, I flatter myself, established my claim to this valuable invention.

It is thus the matter ought to have been expressed by Dr. Roget, who wrote the "Case of Recovery from the Effects of Arsenic," in the 2d volume of "Medico-chirurgical Transactions," rather than by Dr. Marcet, who, it appears, first led his friend to peruse, and, I am sorry to say, *carelessly* peruse, my letter just quoted.

I feel much reluctance thus publicly to accuse either of these gentlemen of wilfully misrepresenting me, and unjustly throwing a veil over *my* efforts with a view to display their own; but, that Dr. Roget's paper has drawn off the public attention from my test towards a mere *modification* of it, if it deserve even that title, is a truth that cannot be disputed.

That this publication has occasioned such a bias in favor of Dr. Marcet, so as to induce a general belief that this gentleman has really produced an entirely *new* test for arsenic, I need go no farther for a proof than the "Critical Analysis" of the last number of your own Journal. In this department of your valuable pages, not only is this supposed new new process displayed to the utmost advantage, but it stands there more free and unconnected with my name, than even Dr. Roget and Dr. Marcet themselves have allowed, for they have vouchsafed so far, as to notice my communication to the Philosophical Magazine.

In another part of your "Analysis," page 70, my test is copied from the "Pharmacologia" of Dr. Ayrton Paris, who also has availed himself of its importance, but without acknowledging its author. This I conceive to be a hard case, while two of my letters upon the subject are to be found in your own Journal. I must beg, however, to correct an error into which you have fallen by copying Dr. Ayrton Paris too closely; the proportions of the arsenic and alkali are totally reversed, so that the experiment must fail if such a process were to be followed.

I fear the mischief will not easily be arrested, for I know Dr. Roget's publication has already influenced men, even of the first character for science and for chemistry. The following is one instance to which I was a witness, and in a situation where I could not with propriety shew my disapprobation by quoting my own letters. It was during one of the public lectures at the Russel Institution, where an example of the test for arsenic was exhibited to a numerous and very respectable audience, and the entire credit of its discovery bestowed upon Dr. Marcet; nor was this omission of my name ever remedied at any subsequent lecture, consequently this assembly was dismissed under a false impression.

I must here acknowledge, that on complaining of this apparent injustice, which I did the same day, I received a very candid and ready confession, and proper explanation of the cause of this omission, from that excellent professor.

I have to complain also that my letter in the "Philosophical Magazine," the only one, by the way, which Dr. Roget has thought prudent to quote in his paper, has been most miserably garbled. There is no such expression in the whole of my short communication, which scarcely occupied one single page in that Journal, as "boiling the suspected matter with solution of potash."

After having noticed, in an obscure way, my process thus perverted, Dr. Roget, and, I should presume, Dr. Marcet, are pleased to say it is "a method of proceeding *analogous* to the one they had described, but which *appears* much less convenient in its practical application."

Now, these gentlemen either do not remember, or they have totally disregarded the two other letters published by me on this subject. Had they looked into the "Medical and and Physical Journal" for May, and also in October, for 1810, they would have seen that I had not confined my test to potass, but had pointed out to your correspondent, the application of *any other alkali.* But I had gone farther in my first letter, for I do not exclude the alkaline earths altogether, though I do not prefer them ; in that letter will be found *lime*-water particularly named ; proving clearly, that, to use *silver* as a test, we must avail ourselves, in some way or other, of the principles of double affinity or elective attraction.

From some palpable incorrections in Dr. Roget's paper, I allude merely to the chemical part, these gentlemen seem to have employed *two* alkalies at the same time, and in the same fluid ; thus, in trying the effect of ammonia and nitrate of silver on Fowler's arsenical solution, in which the arsenic is already fitted for the test, being fully saturated with potass; for this remedy required no ammonia, the nitrate, or indeed any other salt of which silver is the base, might have been presented alone. After such an error I should be tempted to move for a new trial, were my case desperate; I still, however, comfort myself, that, in regard to the validity of my pretensions, I have nothing to apprehend ; and that these gentlemen will candidly allow the priority of my claim to the fullest extent.

I am, Gentlemen,

Your very obedient Servant,

JOS. HUME.

*Long Acre, July* 8, 1812.

